# Dengue Virus Glycosylation: What Do We Know?

**DOI:** 10.3389/fmicb.2017.01415

**Published:** 2017-07-25

**Authors:** Sally S. L. Yap, Terry Nguyen-Khuong, Pauline M. Rudd, Sylvie Alonso

**Affiliations:** ^1^Department of Microbiology and Immunology, Yong Loo Lin School of Medicine, and Immunology program, Life Sciences Institute, National University of Singapore Singapore, Singapore; ^2^Analytics Group, Bioprocessing Technology Institute, A^∗^STAR Singapore, Singapore

**Keywords:** dengue virus, glycosylation, glycomics, E protein, NS1 protein

## Abstract

In many infectious diseases caused by either viruses or bacteria, pathogen glycoproteins play important roles during the infection cycle, ranging from entry to successful intracellular replication and host immune evasion. Dengue is no exception. *Dengue virus* glycoproteins, envelope protein (E) and non-structural protein 1 (NS1) are two popular sub-unit vaccine candidates. E protein on the virion surface is the major target of neutralizing antibodies. NS1 which is secreted during DENV infection has been shown to induce a variety of host responses through its binding to several host factors. However, despite their critical role in disease and protection, the glycosylated variants of these two proteins and their biological importance have remained understudied. In this review, we seek to provide a comprehensive summary of the current knowledge on protein glycosylation in DENV, and its role in virus biogenesis, host cell receptor interaction and disease pathogenesis.

## Dengue Disease, *Dengue Virus* and Dengue Infection Cycle

### Dengue

Dengue (DEN) is an emerging arthropod-borne infectious disease which is caused by DENV. According to the World Health Organization (WHO) (2017), DEN cases have continually increased in recent decades. An estimation of DEN infections worldwide has indicated up to 50–100 million cases per year (WHO, 2017). The virus is transmitted primarily by female *Aedes aegypti* mosquitoes in tropical and subtropical regions. The spread of DEN in non-tropical areas has been associated to the transmission by the secondary vector, *A. albopictus* mosquito which is able to withstand winter temperature (Gould et al., 2010). Four serotypes of DENV (DENV1-4) have been identified to date and co-circulation of these serotypes has been reported in Asia, Africa, and America (Guzman et al., 2010).

Most DENV infections are asymptomatic or remain as mild febrile illness. A classical DEN fever is diagnosed when the patient shows self-limiting high fever, headache, and muscle/joint pain 3–14 days after a mosquito bite. A small proportion of DEN patients may develop DEN hemorrhagic fever and/or DEN shock syndrome (DHF/DSS) which are life-threatening. The clinical manifestations of DHF/DSS include hemorrhagic fever, vascular permeability and plasma leakage, thrombocytopenia and circulatory failure in DSS. To date, there is no specific treatment for DEN and no licensed anti-DENV drug is available. For severe DEN cases, clinical complications are managed by supportive therapy to avoid mortality. The progression to severe DEN (DHF/DSS) has been linked to a phenomenon known as ADE of infection (Halstead, 2014). The ADE hypothesis postulates that during a secondary heterologous DENV infection, preexisting anti-DENV antibodies bind to but fail to neutralize the virus, and promote increased uptake of sub-neutralized virions by Fc-gamma-receptor bearing cells such as DC, macrophages, and monocytes (Kliks et al., 1989; Boonnak et al., 2008). In addition, ligation of Fc receptor stimulates production of Interleukin (IL)-10 which in turn suppresses the cellular anti-viral response (Suhrbier and La Linn, 2003). These events lead to increased viral loads which are believed to correlate with disease severity (Vaughn et al., 2000).

To reduce DEN morbidity and eventually eliminate the disease, an effective vaccine is urgently needed. However, the development of DEN vaccine has been greatly hampered by the potential risk of ADE. The only licensed DEN vaccine (CYD-TDV) available is a tetravalent, recombinant, live attenuated DEN vaccine developed by Sanofi Pasteur (Guy et al., 2015). The vaccine has shown varied efficacy against different serotypes and in different age groups, with safety issues in children below 9 years of age (Capeding et al., 2014; Villar et al., 2015). In addition, large scale efficacy studies have suggested that this vaccine works best in people with pre-existing DENV immunity (Capeding et al., 2014). Thus, WHO recommendations have limited the use of the CYD-TDV vaccine in geographical settings with high DEN burden and in age group 9–45 years old (WHO, 2017). Clearly, while this first-in-human tetravalent DEN vaccine will certainly provide a wealth of knowledge and improve our understanding of immune correlates of protection, a better vaccine is needed to protect the 3.9 billion people that are at risk of DEN infection. Several promising vaccine candidates are currently under development; some have entered the clinical pipeline [reviewed in (Schmitz et al., 2011)]. It is hoped that they will address the shortcomings of the CYD-TDV vaccine.

### *Dengue virus* and Dengue Infection Cycle

DENV belongs to the family *Flaviviridae* of which the members are well known as human pathogens, including WNV, *Zika virus, Yellow fever virus, Tick-borne encephalitis virus*, JEV, and *Hepatitis C virus* (HVC). They are enveloped viruses with positive sense, single stranded RNA and many of them are arthropod-borne viruses. Among all flaviviruses, DENV has the highest impact on global disease burden. The virus particle is about 50 nm in size and the RNA genome (∼10.7 kb) is encapsulated by a protein shell which consists of three structural proteins, namely capsid (C), envelope (E), and (pre)membrane protein (prM/M) (Kuhn et al., 2002).

In order to establish infection, DENV first binds to the host cell receptors via E proteins on the cell surface. The ligand-receptor interaction initiates uptake of the virion through receptor-mediated endocytosis (Acosta et al., 2014). Inside the acidic late endosome, membrane fusion occurs as the virion envelope fuses with the endosomal membrane (Allison et al., 1995; Modis et al., 2004), followed by uncoating of the nucleocapsid and then release of the viral RNA into the cytoplasm. The RNA genome of DENV is translated into a single polyprotein by host ribosomes and is made of three structural (C, E, prM/M) and seven non-structural (NS) (NS1, NS2A/B, NS3, NS4A/B, NS5) proteins. The polyprotein is then cleaved by host and viral proteases to release individual viral proteins (Acosta et al., 2014).

The viral genome replication process within the host cell is mainly driven by the NS proteins. NS1 anchors the replication complex to the ER membrane and interacts physically with NS4B (Youn et al., 2012; Muller and Young, 2013). NS2A is responsible for viral RNA synthesis and virion assembly (Xie et al., 2015). NS3 functions as a serine protease, RNA helicase and nucleotide triphosphatase/RNA triphosphatase, and its protease activity is dependent on the cofactor NS2B (Falgout et al., 1991). NS4A has been reported to induce membrane rearrangement within the host cell, thereby assisting the formation of replication vesicles (Miller et al., 2007). NS5 is a multifunctional enzyme with a methyltransferase (MTase superfamily) domain and a RNA-dependent RNA polymerase domain (Acosta et al., 2014). During virion assembly, the newly synthesized viral RNA interacts with C proteins to form the nucleocapsid. A spiky immature virion is formed when E and prM proteins encounter the nucleocapsid (Yu et al., 2008; Acosta et al., 2014). The maturation process takes place in the trans-Golgi network where prM is cleaved by host furin to generate a smooth surfaced mature virion (Stadler et al., 1997; Yu et al., 2008), which is released into the extracellular environment through the secretory pathway.

## Protein Glycosylation

### What Is Glycosylation?

**FIGURE 1:**
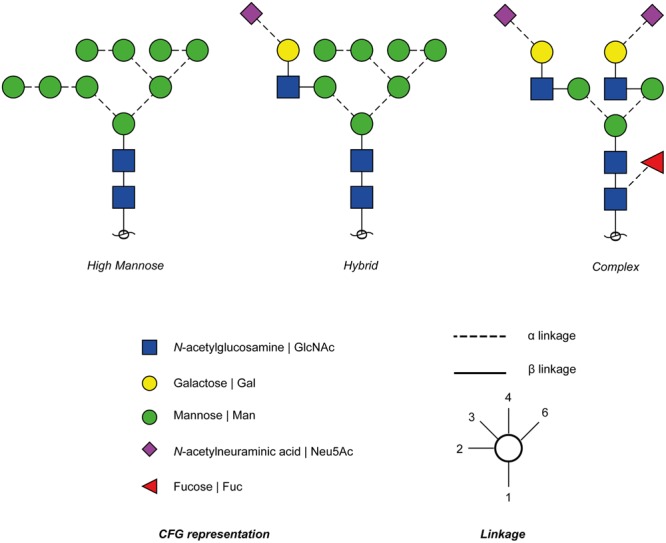
Types of *N*-glycans. High mannose, hybrid and complex N-linked glycans are enzymatically attached to the N-X-S/T sequence of the glycoprotein.

Glycosylation is the post-translational modification of biomolecules such as proteins or lipids through the enzymatic attachment of complex oligosaccharide structures to the peptide backbone or lipid anchor (Varki et al., 2015). Over 70% of the eukaryotic proteome is glycosylated (Dell et al., 2010). The range of complexity of these structures is reflected by their covalent attachment to the protein, the monosaccharide composition of the glycan and combinations of anomeric ring linkages between these monosaccharides. This complexity affects the branching, antennae and topology of the glycan structures, which translates to the overall tertiary and quaternary structure of the glycoprotein. There are two types of protein glycosylation distinguished by their site of attachment on the protein backbone; N-linked glycosylation where the glycan is covalently attached to the asparagine (N) (consensus motif; NxS/T except where x is a proline); and O-linked glycosylation where the glycan is linked to the oxygen from some serine (S) or threonine (T) residues of the protein backbone (Chandler et al., 2013; Varki et al., 2015). N-linked glycans share a common chitobiose core structure. Furthermore, N-linked structures fall under three different classes: (1) high mannose, where the non-reducing composition of the glycans are dominated by mannose sugars that extend from the core, (2) complex, where the branching and extension of the glycans from the core is initiated by *N*-acetyl glucosaminyl transferases; and (3) hybrid structures where the core is extended by both high mannose arm and a complex structure on the other arm (Chandler et al., 2013; Varki et al., 2015) (**Figure 1**).

Such complexity can be found with the isomerism/anomercity of a sugar. Whilst two glycans may have the same composition, the differences of their isomeric linkages can affect the selectivity of their host receptors and thus biology. For example, in the context of avian influenza, the haemagglutinin specifically and exclusively recognizes 2,3-linked sialic acids that are found in the avian host. This is in contrast to 2,6 linked sialic acid normally found in the human host receptors with very low 2,3-linked sialic acid expressed in the lower respiratory tract. Cross reaction between avian H5N1 and such sialic acids caused the recent human epidemics (Shinya et al., 2006; Walther et al., 2013).

### Glycosylation Pathway in Mammalian and Insect/Mosquito Cells

Glycosylation is a highly organized process that involves a network of glycotransferases and glycosidases in the ER-Golgi complex that enzymatically synthesize the glycan as well as trim down the structures so as to achieve the refined structure.

In mammalian cells, *N*-glycosylation takes place predominantly within the ER. Briefly, the initial stages of glycosylation involve the enzymatic synthesis of dolichol-phosphate-NAcGlcNAc_2_Man_9_Glc_3_ which usually takes place across the membrane of the ER. This involves firstly the enzymatic synthesis of dolichol-P-P-GlcNAc_2_Man_5_, before this is “flipped” across the membrane into the ER lumen where further monosaccharides are added (Shi and Jarvis, 2007; Dell et al., 2010). The NAcGlcNAc_2_Man_9_Glc_3_ is transferred to the appropriate NxS/T motif of the protein via the oligosaccharyltransferase as the incipient protein is being translated. As the *N*-glycans transition through the ER-Golgi complex, a series of glycosidases trim down the mannose residues before glycotransferases present in the Golgi extend the antennae of the glycans to produce larger hybrid or complex structures (Varki et al., 2015). In contrast to *N*-glycosylation, *O*-glycosylation occurs entirely in the Golgi apparatus. It does not involve any glyco-lipid intermediates and no glycosidases appear to be involved in their synthesis and processing.

Within the insect kingdom, glycosylation is far simpler yet interestingly insect are able to produce elaborate protein glycosylation in a restricted fashion compared to glycosylation of higher eukaryotes (Rendić et al., 2008). Most our knowledge in insect glycosylation was derived from studies performed on *Drosophila melanogaster* and baculoviral-insect systems. In fact, the bulk of mammalian glycoproteins expressed and/or purified in insect cells have used cell lines from *Spodoptera frugiperda* (sf9, sf21) or *Trichoplusia ni* (High five) (Rendić et al., 2008). Glycoproteins derived from these insect cell systems display glycans that contain predominantly high mannose type structures. However, the long belief that these high mannose and paucimannosidic N-linked structures are the dominant forms in insect-cell derived glycoproteins has been recently challenged by the advent of high resolution glyco-analytical tools which were able to identify glyco-epitopes such as [alpha]1-3 fucosylation (Hsu et al., 1997; Takahashi et al., 1999; Rudd et al., 2000) and double core fucosylated structures ([alpha]1-3 and [alpha]1-6 fucosylation on the core GlcNAc) (Staudacher et al., 1992; Rendic et al., 2006). There were also reports of the extension of the [alpha]1,3-arm of the chitobiose core as opposed to the [alpha]1-6 arm extension found in most mammalian cell lines (Kubelka et al., 1993). Higher complex *N*-glycans can be found in many insect-derived N-linked glycoproteins, however, if grown in serum free media, lysed cell extracts from sf9 and High five cells do lack the nucleotide donors for sialic acid (CMP-NeuAc) (Rendić et al., 2008). Further to this, the presence of a truncated trimannosyl *N*-glycan with an [alpha]1,6-linked fucose was reported (Shi and Jarvis, 2007; Rendić et al., 2008). Extensive work has shown that such structures are the result of the action of an endogenous hexosaminidase specific for the NAcGlcNAc[beta]1,2-Man structure rather than low activity of the [beta]-1, NAc_2_GlcNAc Transferase II responsible for the extension of the mannose arms of complex structures (Kubelka et al., 1994).

Studies performed with mosquito cell lines *A. albopictus* and *A. aegypti* showed that glycoproteins produced in these cell lines display predominantly high mannose and pauci-mannosidic structures (Hsieh and Robbins, 1984). Interestingly, within these initial experiments, the presence of mannosidase-resistant structures was observed (Rhomberg et al., 2006).

### Glycomics

Glycomics as a field has experienced a significant maturation from low to high resolution analysis. In the past decades, scientists have mostly relied on digestive enzymes (Johnson et al., 1994; Mondotte et al., 2007; Hacker et al., 2009), chromatography (Johnson et al., 1994), lectin-binding assay (Johnson et al., 1994; Hacker et al., 2009) and radioactive labeling (Smith and Wright, 1985) to study the glycan structure on glycoproteins. Enzymatic digestion by Endo H and peptide:*N*-glycosidase (PNGase F) remains the most popular method due to its simplicity and allows the investigator to determine whether the glycan structure is asparagine linked (N-linked). In this approach, purified glycoprotein is subjected to specific enzymatic digestion prior to separation on SDS-PAGE. After cleavage of the attached oligosaccharide chains, the digested protein migrates ahead of the undigested form due to a lower molecular weight. Enzymatic digestion of glycoproteins reveals, however, relatively little information on the glycan structure.

Over the recent decade, glycan analysis has dramatically improved through developments in fluorescent labeling, LC and mass spectrometry. There is an abundance of techniques such as LC, CE and mass spectrometry which are available for glycomic analysis. For LC and CE, the field has benefited from labels such as 2-AB, 2-AA (LC separation) and 9-Aminopyrene-1,4,5-trisulfonic acid (CE separation), whereby glycans are tagged at a glycan’s reducing end and the identity of these glycans is assigned based on their retention time behavior across a HILIC or CE, respectively (Rudd and Dwek, 1997; Callewaert et al., 2001). Approaches coupling fluorescence with mass spectrometry (FLR-MS) have helped increase the efficiency of glycomic approaches. In such a platform, the retention time and fluorescence are usually coupled with mass detection to add further confirmation (Houel et al., 2014; Zhang et al., 2016). The use of exoglycosidase arrays adds further confidence to a glycan’s structure elucidation and can help to identify co-eluting glycan species (Marino et al., 2010). The evolving development of glycan labeling has meant that newer, faster labeling and highly sensitive labels such as procainamide or Rapidfluor Mass Spectrometry (RFMS) label can increase the throughput and efficiency of a glycomic analysis.

Various modes of mass spectrometry have been applied to the analysis of released glycans. The most common are MALDI and electrospray ionization. MALDI is a straight-forward method that often requires methods such as permethylation and esterification to increase signal intensity and stabilize labile glycans that contain sialic acids. Electrospray ionization involves a milder desolvation technique and coupled with LC methods and further fragmentation of the molecule, provides high resolution techniques to qualitatively characterize a glycome (Nguyen-Khuong et al., 2015). Detection of glycan fragments which result from fragmentation along the glycosidic bonds (detected in positive mode) and cross-ring (detected in negative mode) (Harvey et al., 2004; Everest-Dass et al., 2012) help to understand the composition and topology of the glycan without the need to adulterate the glycan through derivatization.

Glycoproteomics allows investigators to understand the degree of glycosylation on various sites of the glycoprotein. The platform is adapted from proteomics and as such relies heavily upon mass spectrometry and substantial data analysis. Whilst most glycoproteomic methodologies follow similar approaches to proteomics such as trypsin digestion and analysis, key to any glycoproteomics method is the enrichment of glycopeptides (Mysling et al., 2010; Kolarich et al., 2012). This is important to reduce the ion suppression from peptide mass spectrometry signals without enrichment. This can be performed via HILIC chromatography, in which the enrichment is centered upon exploiting a glycan’s hydrophilicity. Fragmentation data is vital to glycopeptide identification and data analysis must be able to exploit the information which can come from fragmentation modes such as collisionally induced dissociation (CID), high collisional dissociation or electron transfer dissociation/electron transfer high collisional dissociation (ETD/EtHCD) available to the investigator (Scott et al., 2011; Thaysen-Andersen et al., 2016; Stavenhagen et al., 2017). Depending on the strength of fragmentation, information such as glycan composition, site of attachment and peptide backbone are all able to be divulged from a single spectrum (Yang et al., 2016).

### Biological Importance of Glycosylation and Role in Viral Pathogenesis

Glycans either directly or indirectly have diverse biological functions which span but are not limited to inflammation, immunology, infectious diseases, metabolism, embryogenesis, cancer biology and neurodegeneration. At the protein level, glycosylation is responsible for correct protein folding/structure, protein trafficking and stability, receptor/ligand recognition as well as increasing its half-life in the blood stream (Park et al., 2005; Bork et al., 2009; Sumer-Bayraktar et al., 2011; Hart, 2013; Palmisano et al., 2013). At the cellular level, complex sugar structures modulate receptor functions and thus are integral to regulate normal cell–cell, cell-substrate communication and adhesion (Vercoutter-Edouart et al., 2008; Varki et al., 2015). Glycosylation disorders can adversely affect immunity and cancer development. From an immunological perspective, all living cells are covered by a dense glycocalyx and indeed, pathogens and foreign objects must deal with this complex forest of cell surface glycoconjugates upon entering the host (Pickles et al., 2000; Rodrigues et al., 2015).

Viruses do not possess their own glycosylation machinery and by virtue of their opportunistic nature, are heavily dependent upon the glycosylation machinery of the host cell to glycosylate their proteins. HIV, *Influenza virus, Hendra virus, Severe acute respiratory syndrome coronavirus* (SARS-CoV), Hepatitis viruses and WNV are examples of viruses for which glycosylation was shown to be critical to their stability, infectivity and antigenicity (Mir-Shekari et al., 1997; Vigerust and Shepherd, 2007; Medina et al., 2013; Doores, 2015). Firstly, glycosylation can be involved in receptor binding. This is exemplified by HIV and DENV which rely on high mannose type glycosylation to bind to their MRs or DC-SIGN that are present on host immune cells (Cambi and Figdor, 2003; Cambi et al., 2004). Furthermore, glycosylation is required to facilitate proper protein folding and trafficking of the viral membranes using the host chaperones such as calnexin and/or calreticulin proteins (Meunier et al., 1999; Land and Braakman, 2001; Slater-Handshy et al., 2004). Importantly, glycosylation is a means to evade immune recognition within the host by changing glycan sites (Medina et al., 2013), which in turn can increase the diversity of the glycosylation on the virus. In addition, the glycan structure has been reported to mask particular antigenic sites from recognition by neutralizing antibodies (Doores, 2015; Walls et al., 2016).

## Glycoproteins In DENV and Other Flaviviruses

### Envelope (E) Protein

The external protein shell of DEN virion consists of 180 copies of E (53–56 kDa) and prM glycoproteins whereby only E proteins are exposed on the surface (Kuhn et al., 2002). Extensive research over the years has revealed multiple functions of E protein in host receptor attachment, cellular uptake of virion and membrane fusion. E protein forms dimers on the virion surface (Kuhn et al., 2002). The ectodomain of each E monomer without the transmembrane domains and membrane-associated “stem” region displays an elongated structure under Cryo-EM, which is further defined into three distinct domains (Domains I, II, and III) (Rey et al., 1995). The central N-terminal DI separates the dimerization DII from the C-terminal DIII. DIII has been proposed to be the receptor-binding domain (Kuhn et al., 2002) whereby neutralizing monoclonal antibodies against DIII most efficiently block virus initial attachment to mammalian cells (Crill and Roehrig, 2001).

The fusion peptide located at the tip of DII is essential for endosomal membrane fusion and is essential for virus entry (Allison et al., 2001; Kuhn et al., 2002; Huang et al., 2010). Dimerization of E proteins at neutral pH positions the fusion loop into a hydrophobic pocket formed by DI and DIII of the adjacent E monomer. This helps to prevent premature exposure of the fusion loop before endocytosis of the virion by a new host cell. DI forms part of the flexible hinge region which facilitates structural rearrangement of E protein during virion maturation and fusion process (Zhang et al., 2004). Inside the acidic endosome, the pH-dependent hinge at the DI-DII interface (Allison et al., 1995; Modis et al., 2003) allows E dimer to dissociate and rearrange into a trimeric form which serves as a pre-fusion intermediate promoting membrane fusion (Modis et al., 2004).

In the ER lumen of the host cell, membrane-associated E protein is generated after co-translational processing of the viral precursor polypeptide by host Signalase (Acosta et al., 2014). The newly synthesized E protein rapidly heterodimerizes with prM (Lorenz et al., 2002) and three prM-E heterodimers further oligomerize to form a total of sixty heterotrimeric prM-E spikes per subviral particle (Konishi and Mason, 1993; Zhang et al., 2003). This higher-order oligomer has been proposed to represent the preassembly complex (Wang et al., 1999). Translocation of this complex from ER to Golgi is critical as the transition from immature to mature virion is completed only in the trans-Golgi network, where the spiky prM-E trimers are rearranged into 90 flat dimers in a head-to-tail orientation on the virion surface (Kuhn et al., 2002; Zhang et al., 2003).

In the ER, DENV E protein undergoes N-linked glycosylation at two asparagines, N67 and N153 located in DII and DI, respectively (Chambers et al., 1990; Johnson et al., 1994; Hacker et al., 2009). The N67 glycosylation site is unique to DENV and has been proposed to interact directly with DC-SIGN, one of the host cell receptors (Pokidysheva et al., 2006) (see Virus Attachment to Cell Surface and Cell Entry Process). In contrast, N153 (N154 in other flaviviruses) represents the conserved glycosylation site in the family *Flaviviridae*. High resolution crystal structure of *Tick-borne encephalitis virus* E dimer shows the N154-oligosaccharide chain projected overhead of the hydrophobic groove where the fusion loop fits in, suggesting that it functions as an “epitope shield” over the fusion loop to stabilize the dimer contacts (Rey et al., 1995). Consistently, DENV2 and DENV3 mutant viruses lacking N153-glycans due to a single point mutation within the glycosylation motif displayed elevated fusion pH threshold compared to their parental counterpart (Guirakhoo et al., 1993; Lee et al., 1997). The authors proposed that the altered fusion activity of these mutants was likely due to instability of the E dimers.

**FIGURE 2:**
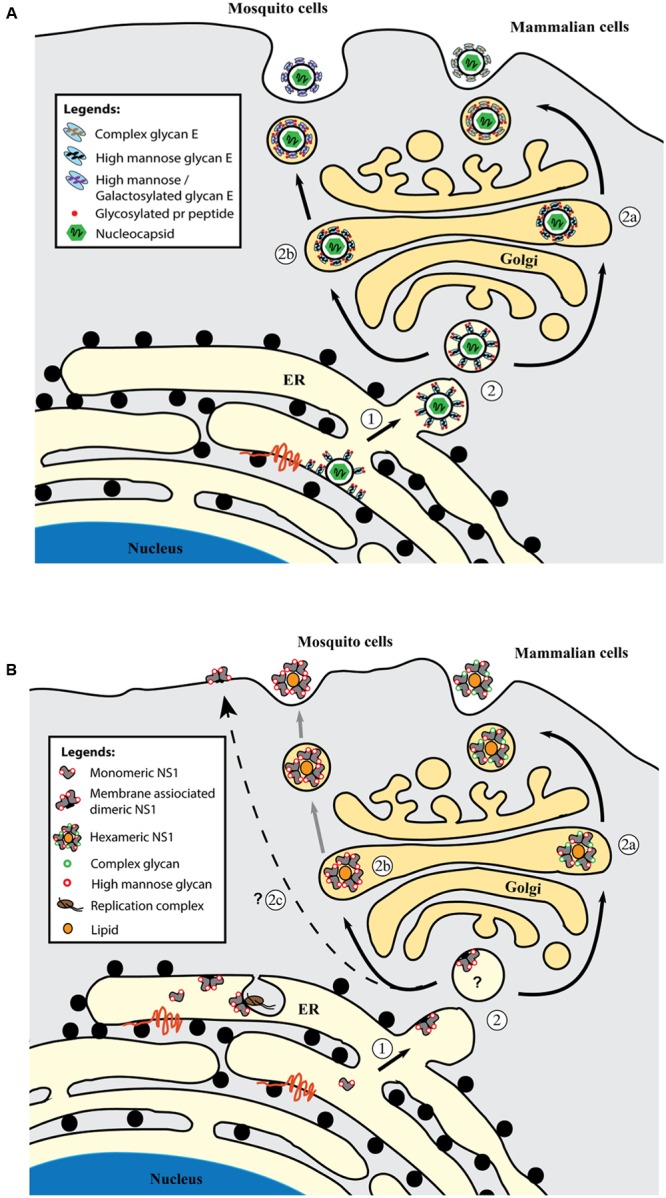
Glycosylation of DENV E **(A)** and NS1 **(B)** proteins in mammalian cells and mosquito cells. **(A)** In ER, newly synthesized E protein is glycosylated and heterodimerizes with prM protein to form a higher order oligomeric preassembly complex. The immature virus particle with prM-E spikes is formed when the nucleocapsid associates with prM-E-rich membranes which buds into the ER lumen (1). The glycans remain of high mannose type on the immature virus particle as it is translocated to Golgi apparatus along the secretory pathway (2). The conformational rearrangement of prM-E spikes and cleavage of prM by host protease furin occurs in the Golgi to produce a mature, smooth virus particle. In mammalian cells, the glycans are further processed and modified into complex glycans before the virus particle is released to the extracellular milieu (route 2a). In mosquito cells, majority of the glycans are high mannose or galactosylated due to the different glycosylation enzymes expressed in insect cells (route 2b). High mannose glycan on the E protein, particularly the N67-glycan facilitates DC-SIGN(+) cell infection and virus propagation. The function of complex glycan on E protein is currently unknown. The glycosylated pr peptides are bound to E protein after furin cleavage and only dissociate at neutral pH in the extracellular milieu. **(B)** Monomeric NS1 protein is glycosylated with high mannose glycans at N130 and N207. The monomer rapidly dimerizes in the ER and membrane-associated NS1 dimers (1) are involved in virus RNA replication. Three NS1 dimers form a soluble hexameric NS1 but the exact location of hexamer formation remains unknown (2). In mammalian cells, the N130 glycans are modified into complex glycans before the soluble NS1 hexamer is secreted out of the cells (route 2a). In mosquito cells, generation of complex glycans doesn’t happen and the lack of complex glycans (N130) on NS1 hexamer affects hexamer stability and greatly reduces its secretion (route 2b). A subset of dimeric NS1 are found on the infected cell surface but the trafficking pathway has yet to be determined (route 2c). High-mannose glycan at N207 stabilizes NS1 dimer.

The glycan structure on DENV E protein has been studied using digestive enzymes (Johnson et al., 1994; Mondotte et al., 2007; Hacker et al., 2009), chromatography (Johnson et al., 1994), lectin-binding assay (Johnson et al., 1994; Hacker et al., 2009) and radioactive labeling (Smith and Wright, 1985). Endo H- and PNGase F-enzymatic digestion revealed the presence of *N*-glycosylation in DENV E protein. No O-linked glycan has been detected to date (Johnson et al., 1994). In mosquito cell-derived virions, the *N*-glycans attached to E protein display heterogeneity in structure and sugar composition where high mannose and paucimannose with terminal mannose residues are the dominant glycoforms (**Figure 2A**) (Smith and Wright, 1985; Johnson et al., 1994; Hacker et al., 2009).

Recently mass spectroscopy has been applied to DENV glycoprotein studies to provide a comprehensive and detailed profile of the glycan moieties (Dubayle et al., 2015; Lei et al., 2015). Using an integrated mass spectroscopy strategy consisting of lectin microarray and MALDI-Time of Flight Mass Spectrometry (MALDI TOF-MS), Lei et al. (2015) have successfully determined the detailed composition of *N*-glycans attached to the E protein from mosquito cell derived mature DENV2. Among the 19 distinct *N*-glycans detected, 15 contain terminal galactosylation while the remaining glycans were identified as high mannose type, complex type, fucosylated and sialylated *N*-glycans. In a separate study, the *N*-glycans from DENV1-4 (vaccine CYD-TDV) produced in mammalian Vero cells have been reported to consist of high mannose, complex and hybrid glycans with complex glycans as the major glycan species (**Figure 2A**) (Dubayle et al., 2015). By performing in-gel proteolysis of E-protein, site specific *N*-glycans have been determined. Sialylated complex glycans and high mannose (6–8 residues) glycans were detected at N153 in all DENV except for DENV2. Besides, most of the complex or hybrid glycans at N153 were found fucosylated. Interestingly, fucosylated glycans were detected only at N153 but not at N67 across all four DENV serotypes. Since high mannose binding DC-SIGN interacts only with N67 glycans on the viral surface (Pokidysheva et al., 2006) and N153-glycan is dispensable for virus production in mosquito and mammalian cells (Bryant et al., 2007), this suggests that N153 glycans may serve a distinct function from N67 glycans in DEN pathogenesis possibly via interaction with an unknown fucose binder or act as a viral glycan shield. For N67 specific glycans, DENV2 was reported to have a different sugar composition from the other three DENV serotypes (Dubayle et al., 2015) whereby a higher content of complex or hybrid glycans was found in DENV2. High mannose glycans were detected as the main glycan species for DENV1, 3 and 4. In addition, sialylated *N*-glycan was detected only in DENV2 at this site. The differential glycosylation pattern between DENV2 and DENV1, 3, 4 may impact on various aspects of dengue pathogenesis including virus tropism, virus fitness, and induction of host responses (see Role of Glycosylation in DENV Life Cycle).

### Non-structural Protein 1 (NS1)

Non-structural Protein 1 (NS1) was first identified as a non-hemagglutinating, soluble complement-fixing antigen in the brain and serum from DENV2-infected mice (Brandt et al., 1970; Smith and Wright, 1985). NS1 has a molecular weight range of 46–55 kDa depending on its glycosylation status. It is a multifunctional glycoprotein which presents in different oligomeric forms and locates at various cellular compartments (Westaway and Goodman, 1987; Flamand et al., 1999).

Non-structural Protein 1 monomer consists of three structural domains namely a β-roll dimerization domain, a wing domain and a β-ladder domain (Akey et al., 2014). The monomer structure is stabilized by six intramolecular disulfide bonds and no intermolecular disulfide bond has been identified in dimeric NS1 (Winkler et al., 1988). However, any one of the three cysteine residues at the C-terminal has been reported to be important for dimer formation (Pryor and Wright, 1993). The β-roll domain and part of the extended wing domain form a hydrophobic protrusion surface that acts as the ER membrane and replication complex (NS4B) interacting site, which is critical for viral RNA replication (Youn et al., 2012; Akey et al., 2014).

NS1 dimer is formed when two β-roll domains dimerize at the center and these dimers tend to trimerize resulting in hexameric NS1 (Flamand et al., 1999; Gutsche et al., 2011; Muller et al., 2012). The NS1 hexamer crystal structure revealed a barrel-shaped oligomer with a central open channel. Three dimers are arranged symmetrically in a way such that the β-roll domains are entirely facing inwards and the channel interior is lined by the hydrophobic protrusion surface contributed by each dimeric component (Akey et al., 2014). The hydrophobic lining allows the NS1 hexamer to be secreted as a lipoprotein whereby the lipid cargo is loaded into the central channel (Gutsche et al., 2011). In contrast to the β-roll domains, glycosylation sites and most of the linear epitopes of NS1 identified are facing outward, representing the most accessible parts of NS1 hexamer by host antibodies (Akey et al., 2014).

Intracellular NS1 is predominantly in dimeric form whereas secreted NS1 is mainly in hexameric form (**Figure 2B**) (Flamand et al., 1999). During protein synthesis, NS1 is cleaved from the viral polypeptide and translocated into the ER lumen. The soluble monomer undergoes dimerization to gain partial hydrophobicity (Flamand et al., 1999), allowing membrane association of NS1 dimer in the absence of a transmembrane domain (Winkler et al., 1989). The exact mechanism of NS1 hexamer formation remains unclear and two possible locations have been proposed including along the Golgi secretory pathway, or immediately after dimerization at the ER (Muller and Young, 2013).

The functions of NS1 are closely associated to its cellular location throughout the virus replication cycle. ER membrane-associated dimeric NS1 has been found to co-localize with viral dsRNA (Mackenzie et al., 1996). Circulating hexameric NS1 is able to bind to the plasma membrane of mammalian cells via the interaction between its *N*-glycans and cell surface glycosaminoglycans, heparin sulfate and chondroitin sulfate E (Avirutnan et al., 2007). Recently, it has been reported that hexameric NS1 contributes to disease pathogenesis of severe DEN (Beatty et al., 2015; Modhiran et al., 2015). The soluble protein acts as a viral toxin that induces pro-inflammatory cytokine response and vascular leakage via Toll-like receptor 4 expressed on immune cells and endothelial cells (Modhiran et al., 2015). Beatty et al. (2015) showed that NS1 vaccination protects mice from NS1-induced vascular leakage which was independent of complement components.

Glycosylation of DENV NS1 occurs right after its cleavage in the ER (Winkler et al., 1988) at two asparagines, N130 and N207 (Putnak et al., 1988; Winkler et al., 1989; Flamand et al., 1999). These two *N*-glycosylation sites are conserved in the family *Flaviviridae*. Recently, a less conserved glycosylation site at N175 has been reported in WNV, *St. Louis encephalitis virus* and *Murray Valley encephalitis virus* but is absent in all four serotypes of DENV (Akey et al., 2014). Intracellular and extracellular DENV NS1 display different types of *N*-glycans as the oligosaccharides undergo modification during the maturation process (Winkler et al., 1989; Pryor and Wright, 1994; Flamand et al., 1999). Intracellular dimeric NS1 *N*-glycans are of high mannose composition regardless of the host cell type (mammalian or mosquito cell) (Mason, 1989). On the other hand, in extracellular hexameric NS1, the N130-glycans consist of complex oligosaccharides whereas the N207-glycans are made of high mannose type sugar chains (Mason, 1989; Pryor and Wright, 1994; Flamand et al., 1999). As dimeric NS1 passes through the Golgi apparatus, two N130-glycans are further modified into the Endo H-resistant, multi-branched complex type before the protein is released (Winkler et al., 1989). The differential modification at these two sites is due to the inaccessibility of N207-glycan by Golgi-resident enzymes after the dimerization of NS1 (Flamand et al., 1999).

### PrM/M Protein

In the DENV replication cycle, prM interacts with E protein and acts as a chaperone to ensure proper E protein folding (Lorenz et al., 2002) and to prevent premature fusion of the virus particle along the secretory pathway by concealing the E fusion loop (Li et al., 2008; Yu et al., 2009). Glycosylation of the prM/M glycoprotein in DENV has not been extensively studied. The protein is glycosylated at N69 (**Table 1**) with circumstantial evidence for N-linked glycosylation at sites 7, 31, and 52 (Courageot et al., 2000). It was found that α-glucosidase inhibitor reduced the amount of prM-E heterodimer, suggesting the *N*-glycans are required for productive folding pathway of these glycoproteins (Courageot et al., 2000). Triglucosylated *N*-glycan at N68 of DENV1 affects the folding of prM by causing a delayed formation of prM-E heterodimer (Courageot et al., 2000).

**Table 1:** Glycosylation of DENV proteins.

Serotypes^∗^	DENV 1	DENV 2	DENV 3	DENV 4
C	NA	NA	NA	NA
PrM±	N64-69			
E	N67 and N153	N67 and N153	N67 and N153	N67 and N153
NS1	N130 and N207	N130 and N207	N130 and N207	N130 and N207
NS2A	Va	Va	Va	Va
NS2B	NA	NA	NA	NA
NS3	Va	Va	Va	Va
NS4A	Va	Va	Va	Va
NS4B	Va	Va	Va	Va
NS5	Va	Va	Va	Va

*NA, not applicable (NxT/S motif is not identified).*

*Va, the presence and number of NxT/S motifs vary among serotypes.*

*^∗^DENV 1 strain Nauru/West Pac/1974 (Accession no:U88535) or strain 45AZ5 (Accession no: NC_001477), DENV 2 strain SG/D2Y98P-PP1/2009 (Accession no: JF327392), DENV 3 strain GZ/10476/2012 (Accession no: KC261634) and DENV 4 isolate GZ30 (Accession no: JQ822247) are aligned against protein entries from Conserved Domain Database (CDD) or UniProt using BLAST (Basic Local Alignment Search Tool).*

*±The *N*-glycosylation site is located at residue 64 to 69 depending on the serotype.*

## Role of Glycosylation In DENV Life Cycle

*N*-glycosylation on both E and NS1 proteins has been shown to play important roles throughout the DENV infection cycle from virion attachment, entry, maturation, assembly to secretion.

### Virus Attachment to Cell Surface and Cell Entry Process

Carbohydrate chains on the DENV E proteins play a critical role in host cell infection at the early step of host receptor binding. Indeed, virus attachment and penetration into mammalian and mosquito cells were blocked by pre-incubation of virus with Concanavalin A, a plant lectin that binds to alpha-linked terminal mannose of high mannose or hybrid glycans (Hung, 1999). Lectins are a group of proteins that recognize carbohydrates through a carbohydrate recognition domain [reviewed in (Zelensky and Gready, 2005)]. To date, various lectin families such as C-type, P-type, L-type, Galectin and Calnexin have been shown to interact with viral components (Liu et al., 2015). C-type lectins are particularly important for DENV infection as they have been shown to be involved in host cell attachment and disease pathogenesis (see Disease Pathogenesis).

The cell membrane-anchored C-type lectin DC-SIGN has been identified as host cell receptor for many viruses (Liu et al., 2015), among which DENV infects DC and monocyte via DC-SIGN (Navarro-Sanchez et al., 2003; Tassaneetrithep et al., 2003). The interaction between DC-SIGN and DENV can be inhibited by the addition of mannose and mannan (Chen et al., 2008) and has been further examined at the molecular level by structural analysis. Cryo-EM data of DENV/DC-SIGN complexes reveals that the carbohydrate recognition domain of DC-SIGN interacts directly with the N67-glycan of E dimers (Pokidysheva et al., 2006). Consistently, lectin (HHA)-resistant DENV which lacks both *N*-glycosylation sites on E protein failed to infect DC-SIGN(+) DC, in contrast to productive infection and replication in DC-SIGN(-) and carbohydrate-independent cells such as Vero, Huh7, C6/36 and Baby Hamster Kidney fibroblasts (BHK-21) (Alen et al., 2012). The presence of N67-glycan on E protein also allows DENV to infect endothelial cells in liver and lymph node via DC-SIGN-related proteins known as DC-SIGNR and L-SIGN, the close homologues of DC-SIGN (Tassaneetrithep et al., 2003; Alen et al., 2012). In addition to DC-SIGN, MR has been identified as another C-type lectin utilized by all four serotypes of DENV to infect macrophages and DC (Miller et al., 2008). Both MR and DC-SIGN bind to DENV E protein with high affinity (*K*_D_ in the sub-nanomolar range) (Lo et al., 2016), despite a different ligand specificity for these two host receptors (Miller et al., 2008). MR shows a preferential binding to terminal mannose, fucose and *N*-acetyl glucosamine while DC-SIGN binds to high-mannose oligosaccharides (Miller et al., 2008). As DC-SIGN and MR have been proposed to be the primary host receptors for DENV during infection (Lo et al., 2016), the engagement to these C-type lectin receptors with diverse glycoforms of E protein may allow DENV to infect a wide range of host cells.

In contrast and interestingly, N67 deglycosylated (N67^-^) DENV1 and DENV2 were found to display enhanced infectivity in mosquito cells (C6/36) compared to wild type (WT) (Ishak et al., 2001; Lee et al., 2010; Alen et al., 2012). For mosquito cells, the entry mode employed by *Flavivirus* (DENV and JEV) was shown to involve membrane fusion instead of receptor-mediated endocytosis (Hase et al., 1989a,b). Hence, it is possible that absence of the N67-glycans from the virion surface reduces steric hindrance and therefore promotes cell membrane attachment and membrane fusion. Finally, N153 deglycosylated (N153^-^) DENV mutant displayed reduced infectivity (10-fold lower) in both mammalian and mosquito cells compared to WT, possibly due to impaired virus entry process (Lee et al., 1997; Hacker et al., 2009), whereby loss of the N153-glycan affected the conformational stability of E proteins and led to premature exposure of the fusion peptide (Yoshii et al., 2013).

### Production of Infectious Virus Particles

#### E Protein Glycosylation

Early studies on DENV E protein showed that *N*-glycosylation is not essential for virus replication in mosquito cells (Bryant et al., 2007; Mondotte et al., 2007). Instead, loss of the N67-glycosylation site through site directed mutagenesis (N67Q) in E protein was sufficient to render DENV2 (strain 16681) growth defective in BHK-21 cells, a DC-SIGN(-) cell line (Bryant et al., 2007). Direct transfection of N67Q mutant RNA into BHK-21 cells neither produced intracellular viral antigen nor released new virus progeny. The lack of virion release may be due to impaired virion secretion along the ER-Golgi secretory pathway in the absence of N67-glycan tag on E protein. However, the same mutant replicated and grew comparably to WT counterpart in C6/36 mosquito cells *in vitro* and in *A. aegypti* mosquito *in vivo* (Bryant et al., 2007), thus supporting that *N*-glycosylation of E protein at position N67 is essential for productive infection in mammalian cells only, consistent with earlier studies (Bryant et al., 2007; Mondotte et al., 2007).

**FIGURE 3:**
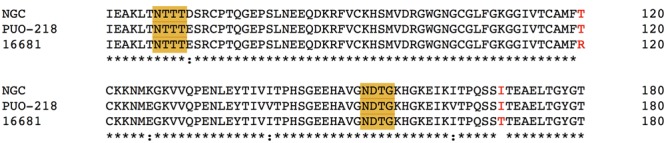
Alignment of E protein sequences from multiple DENV 2 strains. N67 and N153 glycosylation motifs are highlighted in yellow. Strain 16681 differs from strain NGC and PUO-218 at position 120 and 170 (in red). Asterisk indicates positions which have a single, fully conserved residue; colon indicates conservation between groups of strongly similar properties.

Further investigation on the importance of the *N*-glycosylation motif was done by Lee et al. (2010) through extensive point mutation within the conserved N-x-T/S motif of DENV2 (strains PUO-218 and NGC), whereby the conserved residue T69 was replaced with residues of different side chain propensity. Replacement of T69 by a larger and more hydrophobic residue (leucine and valine) either by molecular cloning (Lee et al., 2010) or by passaging the virus under selection pressure (Alen et al., 2012) generated viable virus that retained efficient growth in BHK-21 and Vero cells in the absence of N67 glycan. In addition, N67Q/D mutant virus generated in strain PUO-218 propagated in mammalian cells at reduced growth rate, which is inconsistent with previous studies carried out with DENV2 strain 16681 (Bryant et al., 2007; Mondotte et al., 2007). The differential and virus strain-dependent outcome led to the hypothesis that in the absence of N67 glycosylation, the amino-acid composition of the DII region determines virus survival in mammalian cells (Lee et al., 2010). Multiple sequence alignment showed indeed that strain 16681 differed from strains PUO-218 and NGC at two positions, arginine (R)120 in DII and T170 in DI (**Figure 3**). Uncharged polar threonine replaces charged R120 and hydrophobic isoleucine (I) replaces T170 in strains PUO-218 and NGC. It is possible that the presence of hydrophobic residues facilitates protein folding even without the glycan tag for chaperone-assisted folding and followed by productive protein secretion. Nevertheless, structural comparison of the WT strains and their respective mutants needs to be carried out to confirm this hypothesis.

In contrast to the varying outcomes obtained with N67 mutant viruses and their ability to grow in mammalian cells, it has been consistently reported that these virus variants replicate, propagate in mosquito cells but produce lower virus titers compared to WT (Bryant et al., 2007; Mondotte et al., 2007; Lee et al., 2010).

N153-glycan on E protein is important but not essential for DENV survival in mosquito cells. Successive passages (as low as two passages) of DENV in C6/36 cells *in vitro* resulted in mutation at T155 which ablates the N153-glycosylation motif; however, the non-glycosylated variant was able to propagate in mosquito cells (Lee et al., 1997). N153^-^ DENV grows in both mammalian cells and C6/36 cells and produce lower virus titer than its WT counterpart (Bryant et al., 2007; Lee et al., 2010), which could be due to defective virus budding. Consistently, in a study using transmission electron microscopy, it was shown that virus budding of WT WNV occurs at the plasma membrane while the mature progeny of N154^-^ mutant scatters at the smooth membrane vesicle within swollen ER lumen without budding (Li et al., 2006).

#### NS1 Protein Glycosylation

N130A NS1 mutant virus of DENV1 (Tajima et al., 2008) and DENV2 (NGC) (Pryor et al., 1998) failed to generate viable virus in both mammalian and mosquito cells. Mutation at N130 in DENV4 NS1 caused reduced viral growth in mammalian cells and C6/36 cells (Pletnev et al., 1993). However, N130Q NS1 mutant of DENV2 (16681) produced infectious virus with a similar titer as the WT virus in mammalian cells but with a reduced titer in C6/36 cells (Crabtree et al., 2004). Removal of glycan from N207 in DENV1 and DENV2 (16681) NS1 protein produced similar growth and virus titers compared to WT in mammalian cells despite a delayed cytopathic effect (Crabtree et al., 2004; Tajima et al., 2008), which is not consistent with the observation on DENV2 (NGC) (Pryor et al., 1998). Double mutation attempts (N130Q/N207Q and T132N/T209N) failed to generate genetically stable mutant viruses (Crabtree et al., 2004). Taken together, the findings suggest that at least one of the two *N*-glycosylation sites (probably N130) in NS1 protein is essential to produce viable virus. Similar to E protein, the impact of deglycosylation at this site varies depending on the virus strain and amino acid residue used for replacement.

### NS1 Protein Secretion

In DENV, N130-glycosylation is important but not essential for NS1 secretion in mammalian cells (Despres et al., 1991; Jacobs et al., 1992; Pryor and Wright, 1994; Crabtree et al., 2004). Single and double NS1 mutant proteins are secreted from infected cells even though a reduced secretion yield has been observed (Crabtree et al., 2004; Somnuke et al., 2011). The impact of deglycosylation on secretion is thought to be associated with the stability of the NS1 oligomer. Mutation of N130 or N207 does not affect dimerization of the protein but compromises the stability of the dimer (Winkler et al., 1989; Pryor and Wright, 1994). The dimer appeared more heat sensitive when the *N*-glycan was removed from the protein especially for N207A mutant. The use of tunicamycin, an enzyme targeting the host glycosylation enzymes, allowed confirm that absence of *N*-glycan was solely responsible for the instability of NS1 oligomers instead of changes in the polypeptide backbone in the genetically deglycosylated mutants (Pryor and Wright, 1994; Flamand et al., 1999). Furthermore, the secretion of NS1 was reduced when complex glycans maturation was blocked by glycosylation inhibitors Swainsonine and 1-deoxymannojirimycin (Flamand et al., 1999). Consistently, low levels of NS1 with solely high mannose glycan are secreted from infected mosquito cells, which lack the enzymes to generate complex type glycans. These findings support the proposal that *N*-glycosylation and complex glycan are important for NS1 secretion (Hsieh and Robbins, 1984; Mason, 1989; Thiemmeca et al., 2016). In addition, the majority of the secreted WT and N207Q NS1 proteins are hexamers (Somnuke et al., 2011), whereas secreted N130Q and N130/N207Q NS1 proteins showed reduced hexamer population and increase in higher order oligomer (>675 kDa) population. As compared to mammalian cell-secreted NS1, mosquito cell-secreted NS1 is less stable and undergoes degradation more rapidly at body temperature (Thiemmeca et al., 2016). Hence, the presence of complex glycan at N130 is critical for both NS1 secretion and NS1 hexamer stability (Flamand et al., 1999; Somnuke et al., 2011).

Similarly, it has been proposed that high-mannose glycans at N207 stabilizes NS1 dimer (Pryor et al., 1998; Flamand et al., 1999; Somnuke et al., 2011). Reduced levels of DENV 4 N207Q and N130Q/N207Q NS1 proteins were observed in culture supernatant (Somnuke et al., 2011) which could be explained by two possible scenari: (1) Stability of the secreted mutant forms is compromised due to a different protein conformation (Somnuke et al., 2011). The misfolding of the protein may lead to a less effective secretion of functional hexamer. (2) Transport of the protein from the perinuclear region is affected which in turn compromises the maturation and secretion of the protein (Crabtree et al., 2004).

### Virulence and immunogenicity

Deglycosylated NS1 mutant viruses (N130^-^) are less neurovirulent as evidenced by the reduced mortality observed with mice infected intracranially with the DENV2 and DENV4 mutants (Pletnev et al., 1993; Crabtree et al., 2004). The reduced neurovirulence of these viruses which lack the complex type glycans may be linked to the reduced levels of extracellular hexameric NS1 (Crabtree et al., 2004). The virulence phenotypes observed with N207^-^ mutant viruses varied depending on the DENV strain. DENV2 N207^-^ mutant displayed decreased virulence (Pryor et al., 1998; Crabtree et al., 2004), whereas the DENV4 N207^-^ mutant showed enhanced virulence in mice (Pletnev et al., 1993). The low to undetectable levels of NS1 specific antibodies in mice infected with the DENV4 N207^-^ mutant suggests that the enhanced neurovirulence could be attributed to the reduced immunogenicity of the virus (Pletnev et al., 1993).

### Complement Activation

The complement cascade is the central defense mechanism of innate immunity which triggers the immune effector function to remove infectious pathogens and modified self cells upon activation. The activation and amplification of the complement pathway involves a series of sequential events and the whole process is tightly regulated. Complement can be activated through three major pathways, namely the classical, lectin and alternative pathways [reviewed in (Ricklin et al., 2016)]. The classical pathway is triggered by antibody-antigen complexes whereas the lectin pathway is activated by carbohydrate moieties on the microbial surface. The alternative pathway is activated through direct binding of C3b at the surface of pathogens, which results from the constitutive basal cleavage of C3 (Ricklin et al., 2016).

Mannose binding lectin (MBL) in the lectin pathway triggers antibody-independent activation of complement (Thielens et al., 2002). The proposed MBL-mediated virus elimination mechanisms include (1) direct virus neutralization, (2) C3/C4 deposition on virus surface and (3) interference of host cell lectin receptor binding (Liu et al., 2015). MBL differentiates self- from non-self-antigens based on a sugar density-dependent recognition mechanism (Dam and Brewer, 2010), and the micro pattern of the oligosaccharides structure in addition to the spatial geometry of the macro sugar pattern (Takahashi and Ezekowitz, 2005). It was proposed that the additional N67-glycan in DENV (which is absent in other flaviviruses) could promote a more efficient recognition and binding by MBL (Avirutnan et al., 2011). This hypothesis is supported by improved MBL binding and *in vivo* virus clearance of a genetically engineered WNV with additional N67-glycosylation site (Fuchs et al., 2010). MBL is reactive to high mannose oligosaccharides and thus can efficiently recognize insect cell-derived DENV with high mannose glycans present on its E proteins. Hence, the change of *N*-glycan profile of E protein after one round of replication in mammalian host cells may provide an opportunity to the virus to escape from effective MBL recognition (Fuchs et al., 2011). However, mammalian cell-derived DENV was found to be effectively inhibited and neutralized by mouse MBL (Fuchs et al., 2010). A separate study instead reported preferential binding of recombinant human MBL to insect cell-derived DENV2, whereas virions produced in monocyte-derived DC were not neutralized by human MBL (Avirutnan et al., 2011). It therefore remains unclear whether MBL-mediated virus clearance is optimally engaged during DENV infection in humans.

Furthermore, studies have shown that NS1 interferes with the complement pathway through binding to a number of its components (Muller and Young, 2013). N130Q NS1 was found to bind to C1s proenzyme, C1, C4, and C4b with reduced affinity compared to WT and N207Q NS1 (Somnuke et al., 2011), indicating that the *N*-glycan is required for effective interaction with complement components. The role of NS1 glycosylation in the ability of the protein to interfere with the complement activation, however, has been largely ignored. The *N*-glycans were proposed to be involved in NS1 binding to C4 (Avirutnan et al., 2010) although direct experimental evidence has been missing. A recent study reported that secreted NS1 binds directly to C4BP, a major inhibitor of the C4b component (Thiemmeca et al., 2016). This binding leads to the recruitment of C4BP on cell surface via NS1 and inactivates C4b thereby interfering with the formation of the membrane attack complex (MAC). Furthermore, the work has demonstrated a competitive binding of NS1 to MBL, which prevents MBL-mediated DENV destruction. The presence of secreted NS1 in the saliva of *Aedes* mosquito suggests that secreted NS1 protein could help DENV to escape the host innate immune surveillance during virus transmission (Thiemmeca et al., 2016).

### Disease Pathogenesis

Severe DEN (DHF/DSS) is characterized by increased vascular permeability and plasma leakage, thrombocytopenia, hemorrhagic fever and circulatory failure in DSS (WHO, 2017). The current paradigm proposes that viral-induced pro-inflammatory cytokine storm drives the disease progression to DHF/DSS (Pang et al., 2007). Direct interaction between DENV and CLEC5A expressed on macrophages indicates that virus glycosylation plays a role in DEN pathogenesis (Chen et al., 2008; Wu et al., 2013). Similar to the engagement to DC-SIGN, DENV binding to CLEC5A relies on sugar moieties and can be inhibited by exogenous fucose and mannose (Chen et al., 2008). However, CLEC5A binding does not mediate viral entry into the host cell, instead it serves as a cooperative signaling receptor to MR/DC-SIGN that activates macrophage inflammasome and triggers the production of pro-inflammatory cytokines (Chen et al., 2008; Wu et al., 2013; Lo et al., 2016). Consistently, anti-CLEC5A monoclonal antibody reduced DENV-induced vascular leakage in a mouse model (Chen et al., 2008), which further supports that targeting the viral glycoprotein-host lectin receptor interactions represents a potential therapeutic approach to counteract the excessive inflammatory responses involved in severe DEN.

## Future Perspectives and Conclusion

Glycosylation is a post-translational modification which significantly affects the conformation of a protein. It is a heterogeneous process that is highly host-cell specific. Viruses have evolved to utilize their host’s glycosylation machinery so as to optimize their fitness, infectivity, replication and virulence.

The role of glycosylation and glycan structures in DENV virulence has yet to be reported with evidence of attenuated phenotypes in symptomatic mouse models. Given the impact of glycosylation in virus entry and virus fitness in mammalian cells, it is highly likely that deglycosylated DENV mutants will display reduced virulence *in vivo*. The ability of Celgosivir treatment, a bicyclic iminosugar that inhibits glycosylation through negatively binding to ER [alpha]-glucosidase II, to protect mice from a lethal DENV challenge indirectly demonstrates the importance of glycosylation in DENV virulence (Perry et al., 2013; Sayce et al., 2016; Warfield et al., 2016).

To date, out of the eight DENV potential glycoproteins (**Table 1**), only E and NS1 proteins have been characterized from a glycosylation standpoint and not across all the DENV serotypes. Despite the biological importance of these structures being recognized, efforts to characterize the nature of the glycan structures in DENV have remained timid. There is for example little understanding of how glycosylation impacts DENV cell tropism where different glycan variants may influence binding of DENV to various host cell receptors and subsequent cell infection. Characterization of DENV glycoforms has been mainly performed in the mammalian cell line BHK-21 but no study has been conducted in more relevant primary mammalian cell types including Langerhans cells, monocytes, hepatocytes, and endothelial cells. Furthermore, in-depth characterization of the glycan structures using the latest glycomics technologies has yet to be reported for DENV. Associating the contribution of these glycans to the structure and ultimately function of the virion glycoproteins indeed requires glycomics and glycoproteomics. Such data need to be modeled using computational approaches as methods for crystallizing glycoproteins remains a complicated feat.

Furthermore, the role of glycosylation is also very important to recognize in biotherapeutic strategies. While substantial efforts have been devoted to developing neutralizing antibodies against DENV, the potency of these antibodies is largely dictated by the accessibility of the epitope that they target which can be influenced by the glycosylated status of the protein (Smith et al., 2013). Consistently, E protein glycosylation site has been reported to modulate the binding of neutralizing antibodies against a highly conserved, serotype cross-reactive epitope (Dejnirattisai et al., 2015). These challenges have prompted substantial investment into elucidating the three-dimensional conformation of the protein-antibody complexes and more importantly how glycosylation contributes to the tertiary and quaternary arrangements of the different glycoproteins on the virion. This approach is critical for the development of new therapeutics with broader activity and increased efficacy.

In conclusion, the DEN field as a whole would benefit greatly from in-depth understanding and characterization of the glycosylation patterns of DEN virions. With the recent technical advances in the fields of glycomics and glycoproteomics, this has become possible and will depend on productive interactions between glycobiologists and DEN virologists.

## Author Contributions

SY, TN-K, and SA wrote the manuscript. PR provided suggestions and edited the manuscript.

## Conflict of Interest Statement

The authors declare that the research was conducted in the absence of any commercial or financial relationships that could be construed as a potential conflict of interest.

## Abbreviations

ADE: antibody-dependent enchancement
C: capsid
CE: capillary electrophoresis
CLEC5A: C-Type Lectin Domain Family 5 Member A
Cryo-EM: cryoelectron microscopy
DC: dendritic cell
DC-SIGN: dendritic cell-specific Intercellular adhesion molecule-3-Grabbing Non-intergrin
DEN: dengue
DENV: *Dengue virus*
DHF/DSS: dengue hemorrhagic fever/shock syndrome
DI: domain I
DII: domain II
DIII: domain III
E: envelope
Endo H: endoglycosidase H
ER: endothelium reticulum
HILIC: hydrophilic interaction chromatography
HIV: *Human immunodeficiency virus*
JEV: *Japanese encephalitis virus*
LC: liquid chromatography
MALDI: Matrix Assisted Laser Desorption Ionization
MR: mannose receptor
NS: non-structural
PNGase F: peptide:*N*-glycosidase
prM/M: (pre)membrane
WNV: *West Nile virus*
